# Subcellular regulation of glucose metabolism through multienzyme glucosome assemblies by EGF–ERK1/2 signaling pathways

**DOI:** 10.1016/j.jbc.2022.101675

**Published:** 2022-02-02

**Authors:** Miji Jeon, Krishna M. Chauhan, Gregory L. Szeto, Minjoung Kyoung, Songon An

**Affiliations:** 1 Department of Chemistry and Biochemistry, University of Maryland Baltimore County (UMBC), Baltimore, Maryland, USA; 2 Department of Chemical, Biochemical and Environmental Engineering, University of Maryland Baltimore County (UMBC), Baltimore, Maryland, USA; 3 Program in Oncology, Marlene and Stewart Greenebaum Comprehensive Cancer Center, University of Maryland, Baltimore, Maryland, USA

**Keywords:** glucose metabolism, protein assembly, extracellular-signal-regulated kinase (ERK), epidermal growth factor (EGF), metabolic regulation, glycolysis, serine biosynthesis, live-cell imaging, metabolic condensate, ATCC, American Type Culture Collection, dFBS, dialyzed fetal bovine serum, DMSO, dimethyl sulfoxide, EGF, epidermal growth factor, EGFR, EGF receptor, ERK, extracellular signal-regulated kinase, mEGFP, monomeric enhanced GFP, PFKL, liver-type phosphofructokinase 1, PHGDH, phosphoglycerate dehydrogenase, shRNAs, short-hairpin RNAs

## Abstract

A multienzyme metabolic assembly for human glucose metabolism, namely the glucosome, has been previously demonstrated to partition glucose flux between glycolysis and building block biosynthesis in an assembly size-dependent manner. Among three different sizes of glucosome assemblies, we have shown that large-sized glucosomes are functionally associated with the promotion of serine biosynthesis in the presence of epidermal growth factor (EGF). However, due to multifunctional roles of EGF in signaling pathways, it is unclear which EGF-mediated signaling pathways promote these large glucosome assemblies in cancer cells. In this study, we used Luminex multiplexing assays and high-content single-cell imaging to demonstrate that EGF triggers temporal activation of extracellular signal-regulated kinases 1/2 (ERK1/2) in Hs578T cells. Subsequently, we found that treatments with a pharmacological inhibitor of ERK1/2, SCH772984, or short-hairpin RNAs targeting ERK1/2 promote the dissociation of large-sized assemblies to medium-sized assemblies in Hs578T cells. In addition, our Western blot analyses revealed that EGF treatment does not increase the expression levels of enzymes that are involved in both glucose metabolism and serine biosynthesis. The observed spatial transition of glucosome assemblies between large and medium sizes appears to be mediated by the degree of dynamic partitioning of glucosome enzymes without changing their expression levels. Collectively, our study demonstrates that EGF–ERK1/2 signaling pathways play an important role in the upregulation of large-sized glucosomes in cancer cells, thus functionally governing the promotion of glycolysis-derived serine biosynthesis.

Glycolysis is an essential metabolic process for not only energy production but also a number of cellular functions in cells. We have previously reported the identification of a multienzyme metabolic assembly for glucose metabolism, namely the glucosome, which is comprised of at least four pathway enzymes, including human liver-type phosphofructokinase 1 (PFKL), human liver-type fructose 1,6-bisphosphatase, pyruvate kinase muscle-type 2, and phosphoenolpyruvate carboxykinase 1 (1). When glucosomes are visualized with a fluorescent protein tag in human cancer cells, particularly including human breast carcinoma cancer cells (Hs578T), they are displayed in three different sizes under fluorescence live-cell microscopy (1) namely, small-, medium-, and large-sized glucosomes. Small-sized glucosomes are defined to have less than our calculated area of a point spread function for the emission of monomeric enhanced GFP (mEGFP) (*i.e.*, ∼0.1 μm^2^). Medium-sized glucosomes are then defined to range from 0.1 μm^2^ to 3 μm^2^ in size because, unlike in cancer cells, glucosomes that are larger than 3 μm^2^ in size are not detectable in noncancerous human breast cells (Hs578Bst) from our conditions (1). Accordingly, large-sized glucosomes have larger than 3 μm^2^ in size that we have detected only in human cancer cells (1). Moreover, we have revealed that the supplement of epidermal growth factor (EGF) in a growth medium promotes the formation of large-sized glucosomes to accommodate glucose flux being channeled into serine biosynthesis in Hs578T and HeLa cells (1, 2). Nonetheless, it has remained elusive which EGF-triggered signaling pathways regulate the assembly of large-sized glucosomes in cancer cells.

Epidermal growth factor is known to be an important element for cell growth and proliferation (3, 4). Specifically, the EGF receptor (EGFR), a transmembrane protein tyrosine kinase, is frequently mutated and overexpressed in various types of human cancers, including breast cancers (4). Indeed, EGF-mediated activation of EGFR has been shown to stimulate a number of signaling pathways (5, 6, 7, 8, 9, 10). Of particular, the EGF-associated signaling pathways that include SRC family kinases, extracellular signal-regulated kinases (ERKs), and protein kinase B (AKT) pathways have been functionally associated with glucose metabolism and serine biosynthesis, for example, by driving the activation of PFK (11, 12, 13), hexokinase (9, 14) and phosphoglycerate kinase 1 (15) or the inhibition of pyruvate kinase (8, 16) in various cancer cells, as well as by promoting the upregulation of phosphoglycerate dehydrogenase (PHGDH) particularly in breast cancer cells (16, 17). However, it has not been clear whether only one or a few selective kinases and their effectors or a collective response from all of these factors play a role in regulation of glucose flux and thus their contribution to cancer cell metabolism.

In this work, we investigate which downstream kinase-associated pathway is activated by EGF to spatially regulate glucosome dynamics and thus functionally control glucose flux in cancer cells. We have firstly used a Luminex multiplexing assay and found that ERK1/2 is significantly activated from Hs578T cells in the presence of EGF. Subsequent pharmacological inhibition, genetic knockdown, and high-content single-cell imaging analyses have further corroborated that EGF–ERK1/2 signaling pathways specifically regulate the assembly of large-sized glucosomes in Hs578T cells. Western blots are also performed to assess the effects of the EGF treatment on enzymes’ expression levels and thus spatial dynamics of glucosomes. Taken all together, we provide compelling evidence that EGF–ERK1/2 signaling pathways are responsible for upregulating the formation of large-sized glucosomes in cancer cells, thus promoting glucose flux into serine biosynthesis for cancer cell metabolism.

## Results

### Epidermal growth factor triggers temporal activation of ERK1/2 in Hs578T cells

Previously, we have demonstrated that various sizes of multienzyme metabolic assemblies, glucosomes, are formed in the cytoplasm of human cancer cells, including Hs578T cells, by enzymes that catalyze rate-determining irreversible steps in glycolysis and in gluconeogenesis (1). Particularly, the subpopulation of Hs578T cells showing large-sized glucosomes was significantly increased by ∼ 46% when the cells were continuously exposed to 30 ng/ml EGF for at least 2 weeks (1). Meanwhile, EGF-treated cancer cells have shown to promote glycolysis-derived serine flux particularly in breast cancer cells (7, 16, 17, 18). Accordingly, we have proposed that large-sized glucosomes are spatially regulated to functionally accommodate the promotion of glycolysis-derived serine flux in breast cancer cells (1), which is further corroborated by our mathematical modeling study (2). However, due to the myriad roles of EGF in cancer cell signaling (4, 19), it has remained elusive which EGF-stimulated signaling pathways are responsible for spatially and thus functionally regulating glucosome dynamics, particularly for the increased utilization of large-sized glucosomes, in human cancer cells.

**Figure 1:**
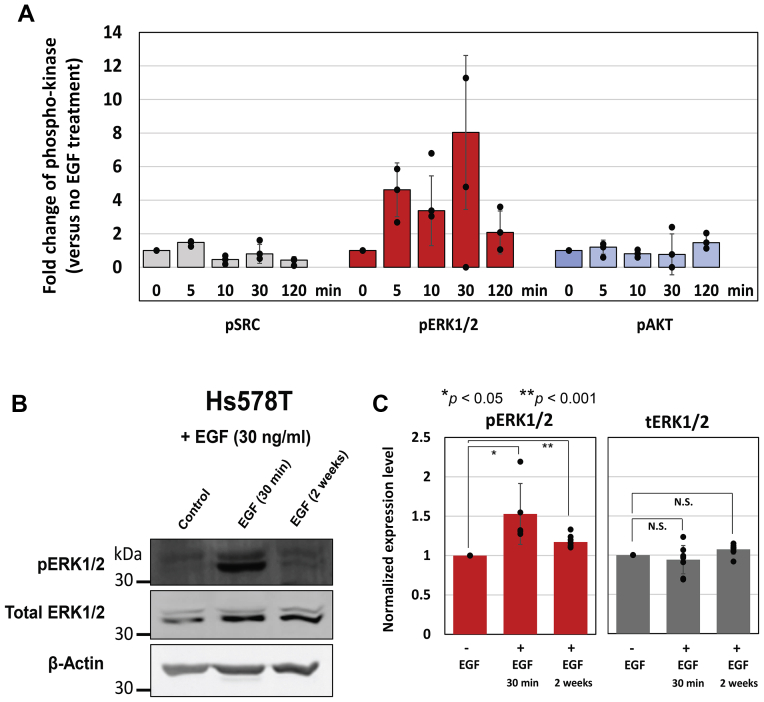
**EGF-stimulated activation of ERK1/2.***A*, epidermal growth factor-treated Hs578T cells showed a significant increase of ERK1/2 activation in various time points relative to SRC and AKT kinases. Note that the levels of phosphorylation on other screened kinases were similar or lower than the phospho-kinase levels detected for SRC and AKT. Epidermal growth factor-untreated Hs578T cells were used as a baseline control. The data reported here is from at least three duplicated independent trials. *B*, Western blot analysis also showed the activation of ERK1/2 in the presence of EGF (30 ng/ml) from Hs578T cells. *C*, expression levels of total ERK1/2 (tERK1/2) and phosphorylated ERK (pERK1/2) were normalized based on load controls (β-actin), respectively. The error bars represent the SDs of at least three independent experiments. Statistical analyses were performed using two-sample two-tailed *t* test. ∗*p* < 0.05, ∗∗ *p* < 0.001. N.S.; not significant. EGF, epidermal growth factor; ERK, extracellular signal-regulated kinase.

In this work, we have first investigated which signaling pathways EGF activates in Hs578T cells where large-sized glucosomes are spatially and temporally promoted. Because many protein kinases have been implicated for EGFR signaling pathways, we have carried out Luminex multiplexing assays to screen a set of EGF-associated signaling kinases that are proposed to control glucose metabolism and/or serine biosynthesis in various cancer cells (5, 6, 7, 8, 9, 10, 19). Briefly, Hs578T cells were lysed after addition of 30 ng/ml of EGF in our growth conditions (*i.e.*, RPMI 1640 and 10% dialyzed fetal bovine serum (dFBS)). As a control, the cells were also grown in the same growth medium without EGF. Lysates of Hs578T cells were screened for a total of 20 kinase-specific antibodies, which particularly include phospho-kinase antibodies of SRC, AKT, and ERK1/2. Our multiplexing assays revealed that when Hs578T cells were treated with EGF for 5 to 120 min, gradually increased phosphorylation of ERK1/2 was detected and the level of phospho-ERK1/2 was peaked at 30 min by ∼ 8-fold higher than the level of phospho-ERK1/2 from EGF-untreated Hs578T cells (Fig. 1*A*). The increased level of phospho-ERK1/2 after 30 min incubation with EGF was further confirmed by Western blots (Fig. 1, *B* and *C*). However, none of the other kinases we screened, including SRC and AKT kinases, showed significant changes within the observation time (Fig. 1*A*). At the same time, our multiplexing assays also indicated that there was hardly distinguishable change in the phosphorylation levels of all the tested kinases, including ERK1/2, when Hs578T cells were treated with EGF continuously for 2 weeks. However, when we carried out Western blots, the level of phospho-ERK1/2 in the EGF-treated cells for 2 weeks was higher than its level from the EGF-untreated cells (Fig. 1, *B* and *C*). Therefore, these results support that EGF triggers temporal activation of ERK1/2 but appear to uphold a sustained level of phosphorylated ERK1/2 to promote large-sized glucosomes in Hs578T cells.

### Inhibition of ERK1/2 promotes the dissociation of large-sized glucosomes in Hs578T cells

**Figure 2:**
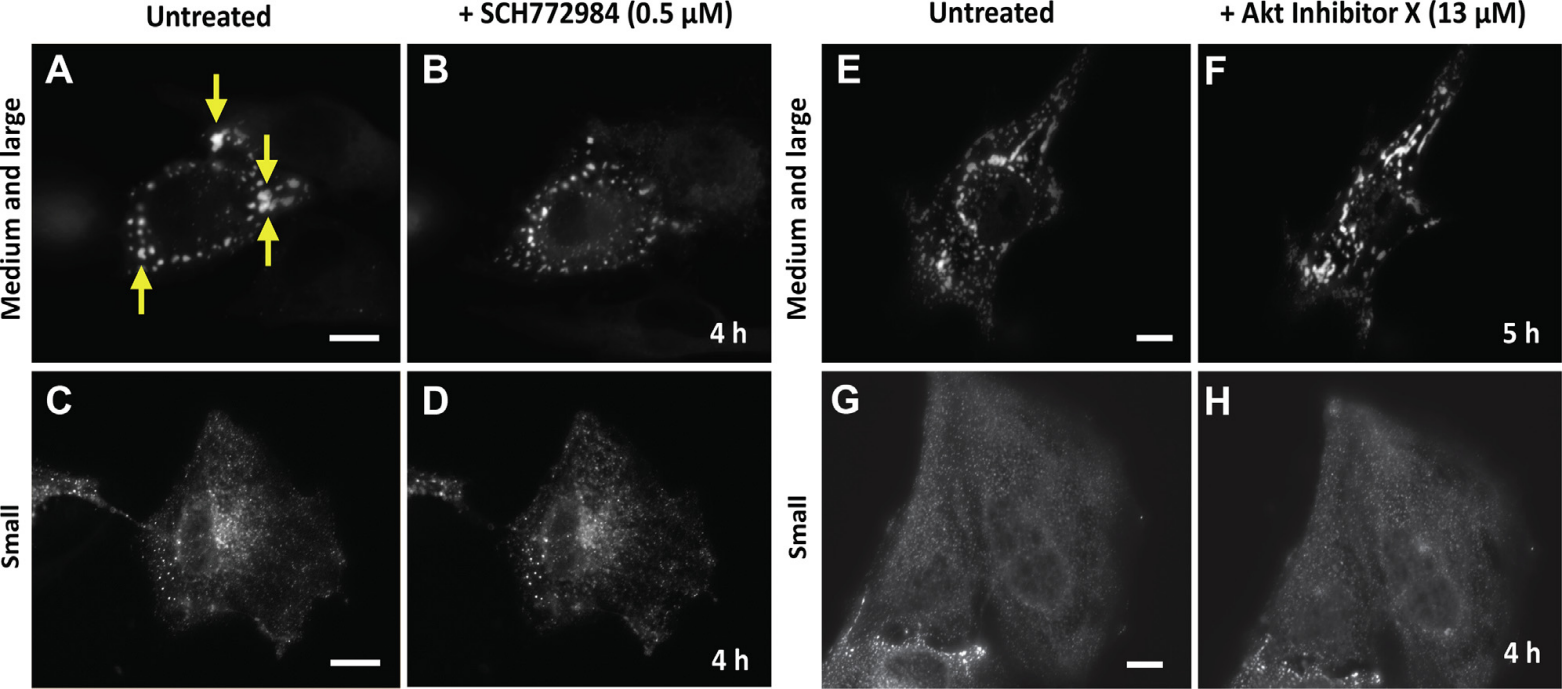
**Pharmacological inhibition of ERK1/2 and AKT in Hs578T cells.***A*–*D*, representative Hs578T cells expressing PFKL-mEGFP in medium and large sizes (*A* and *B*) or in small sizes (*C* and *D*) were imaged before (*A* and *C*) and after (*B* and *D*) the addition of the ERK1/2 inhibitor, SCH772984 (0.5 μM). The *yellow arrows* in (*A*) indicate some of the large-sized glucosomes that were dissociated after 4 h treatment of SCH772984. The scale bars represent 10 μm. *E*–*H*, as negative controls, representative Hs578T cells expressing PFKL-mEGFP in medium and large sizes (*E* and *F*) or in small sizes (*G* and *H*) were imaged before (*E* and *G*) and after (*F* and *H*) the addition of the AKT inhibitor, AKT Inhibitor X (13 μM). The scale bars represent 10 μm. ERK, extracellular signal-regulated kinase; mEGFP, monomeric enhanced GFP; PFKL, liver-type phosphofructokinase 1.

**Figure 3:**
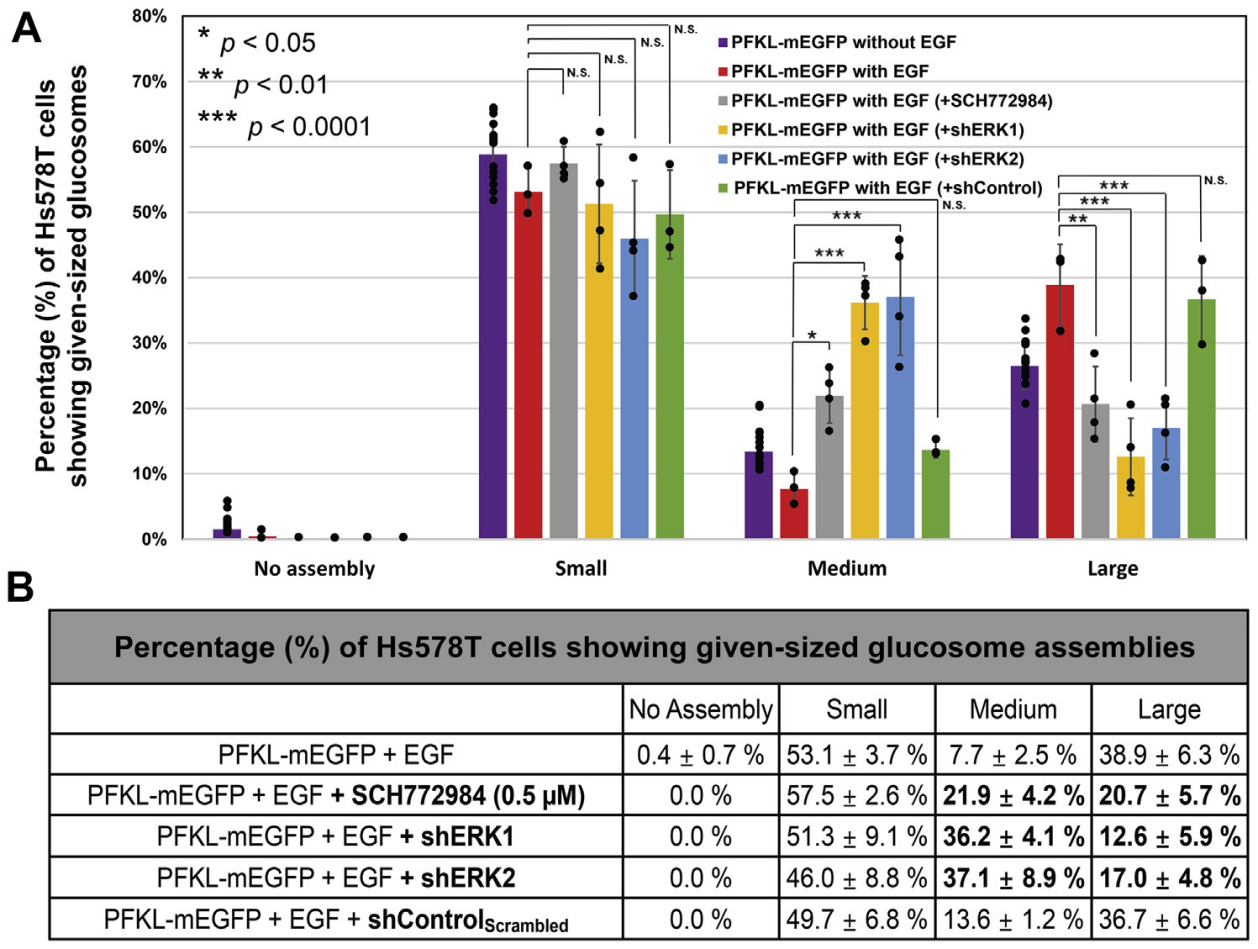
**High-content imaging analysis of glucosomes from Hs578T cells.***A*, the percentages (%) of EGF-treated Hs578T cells displaying each size of PFKL-mEGFP assemblies were quantified from at least three independent imaging sessions in the presence of SCH772984 (*gray*), shERK1 (*yellow*), shERK2 (*blue*), or shControl_Scrambled_ (*green*). Note that the previously reported distributions of glucosomes in various sizes in the absence and presence of 30 ng/ml EGF (*purple* and *red*, respectively) (1) are also graphed together for direct comparison. *B*, a table shows the average percentages (%) of Hs578T cells displaying the given sized glucosomes at each condition along with their SDs (±). Statistical analyses were performed using Tukey’s multiple comparison tests for two-way ANOVA analysis. ∗*p* < 0.05, ∗∗ *p* < 0.01, ∗∗∗ *p* < 0.0001. N.S.; not significant. EGF, epidermal growth factor; mEGFP, monomeric enhanced GFP; PFKL, liver-type phosphofructokinase 1.

Accordingly, we hypothesized that EGF–ERK1/2 signaling pathways were responsible for regulating large-sized glucosomes in cancer cells. To evaluate this hypothesis, we performed fluorescence live-cell imaging to track real-time changes of large-sized glucosomes by expressing mEGFP-tagged PFKL (PFKL-mEGFP) as a glucosome marker in the presence of a small-molecular ERK inhibitor. In this work, we have used SCH772984, an ATP-competitive ERK1/2 inhibitor, because it is found to not only inhibit the kinase activity of ERK1/2 but also block ERK1/2 being phosphorylated by its upstream effectors (20, 21, 22). Noticeably, pharmacological inhibition of ERK1/2 with 0.5 μM of SCH772984 disrupted and thus dissociated large-sized glucosomes in EGF-treated Hs578T cells (Fig. 2, *A* and *B*). In addition, high-content imaging analyses confirmed that the population of Hs578T cells showing large-sized glucosomes was significantly decreased ∼47% (*i.e.*, from 38.9% to 20.7%) in the presence of 0.5 μM SCH772984, whereas the population of Hs578T cells showing medium-sized glucosomes was concurrently increased ∼184% (*i.e.*, from 7.7% to 21.9%) in the presence of 0.5 μM SCH772984 (Fig. 3, red *versus* gray). However, there was no considerable change of the population of Hs578T cells that initially showed small-sized glucosomes (*i.e.*, 53.1% to 57.5%) (Figs. 2, *C* and *D* and 3). As a control, we also inhibited the kinase activity of AKT with a cell-permeable, potent small-molecule inhibitor (*i.e.*, Akt Inhibitor X) (23) because AKT has been implicated to control the enzymatic activity of PFKL through EGF-triggered signaling pathways (11, 12, 13). However, at least in our experimental conditions, the treatment of Akt inhibitor X showed no significant change in terms of preferential utilization of specific sizes of glucosomes in Hs578T cells (Fig. 2, *E*–*H*). Along with a negative vehicle [*i.e.*, dimethyl sulfoxide (DMSO)] control, our data collectively support a notion that EGF–ERK1/2 signaling pathways indeed play an essential role in the regulation of large-sized glucosomes in cancer cells.

### Knockdown of ERK1/2 also reduces the population of Hs578T cells showing large-sized glucosomes

**Figure 4:**
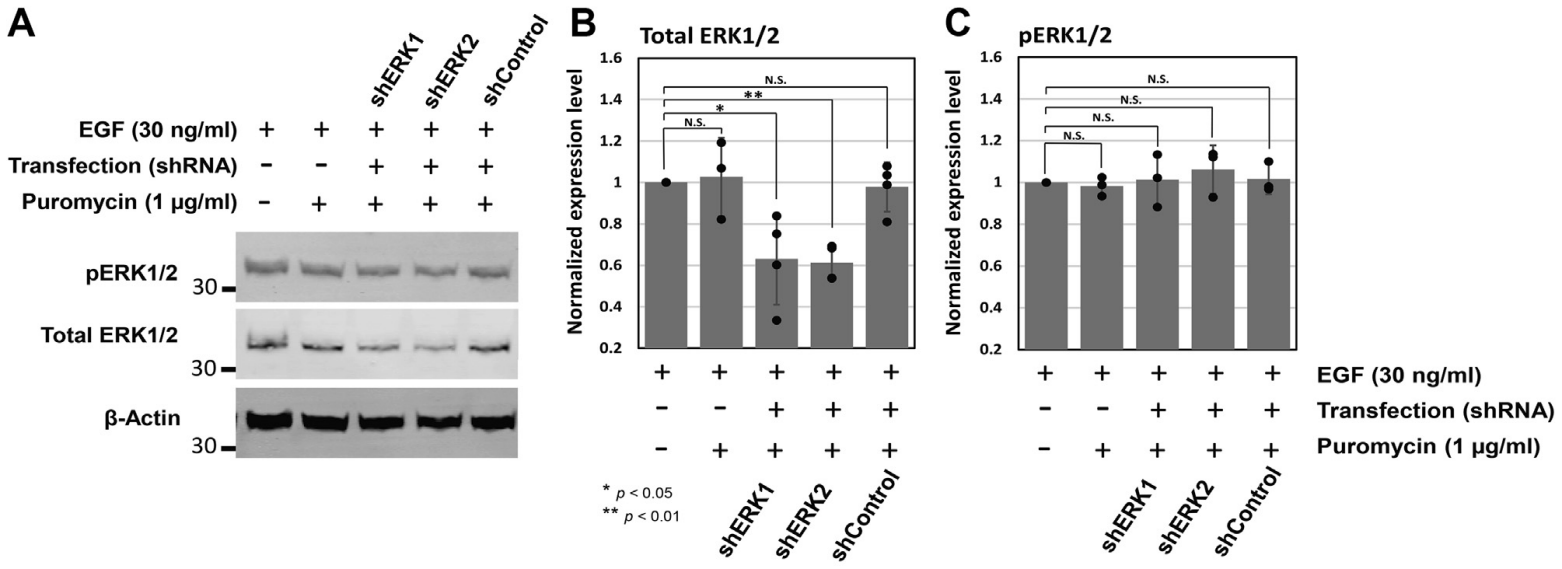
**Western blot analysis of ERK1/2 knockdown.** Epidermal growth factor-treated Hs578T cells were transfected with shERK1, shERK2, or shControl_Scrambled_ and subsequently selected in the presence of puromycin (1 μg/ml). *A*, Western blot analysis showed the knockdown of total ERK1/2, but no change was detected for pERK1/2 in the presence of shERK1/2. *B* and *C*, expression levels of total ERK1/2 and phosphorylated ERK1/2 (pERK1/2) were normalized based on load controls (β-actin), respectively. The error bars represent the SDs of at least three independent experiments. Statistical analyses were performed using two-sample two-tailed *t* test. ∗*p* < 0.05, ∗∗ *p* < 0.01. N.S.; not significant. ERK, extracellular signal-regulated kinase.

To validate our pharmacological inhibition study (Figs. 2 and 3-gray), we also knockdowned the expression level of ERK1/2 using validated short-hairpin RNAs (shRNAs) against ERK1/2 (*i.e.*, shERK1 or shERK2) (24). Briefly, after we dually transfected Hs578T cells with PFKL-mEGFP and shERK1 or shERK2, we subsequently treated the cells with puromycin (1 μg/ml) to select dually transfected cells for further analysis. After confirming on average ∼37 to 39% knockdown of ERK1/2 in the presence of shERK1/2 by Western blots (Fig. 4), we performed our high-content imaging analysis. Indeed, genetic knockdown of ERK1 and ERK2 decreased ∼68% (*i.e.*, from 38.9% to 12.6%) and ∼56% (*i.e.*, from 38.9% to 17.0%) of the Hs578T cells showing large-sized glucosomes, respectively (Fig. 3, red *versus* yellow and blue). At the same time, the population of Hs578T cells showing medium-sized glucosomes was drastically increased from 7.7% to 36.2% and 37.1% in the presence of shERK1 and shERK2, respectively (Fig. 3, red *versus* yellow and blue). Our two-way ANOVA analysis revealed that the impacts of ERK1 or ERK2 knockdown on the populations of Hs578T cells showing medium- and large-sized glucosomes (Fig. 3, from red to yellow or blue, *p* < 0.0001 for ERK1 or ERK2 knockdown) were more significant than what we observed with the ERK1/2 inhibitor, SCH772984 (Fig. 3, from red to gray, *p* < 0.05 or *p* < 0.01, respectively). Along with the no-shRNA control, we also confirmed that dually transfected cells expressing PFKL-mEGFP and shControl_Scrambled_ did not show any significant change of glucosome utilization in Hs578T cells (Fig. 3, red *versus* green). Collectively, these results further assure that EGF–ERK1/2 signaling pathways are tightly associated with spatial dynamics of glucosomes in large and medium sizes in Hs578T cells.

### Epidermal growth factor treatment show no increased levels of enzymes in glucose metabolism and serine biosynthesis

**Figure 5:**
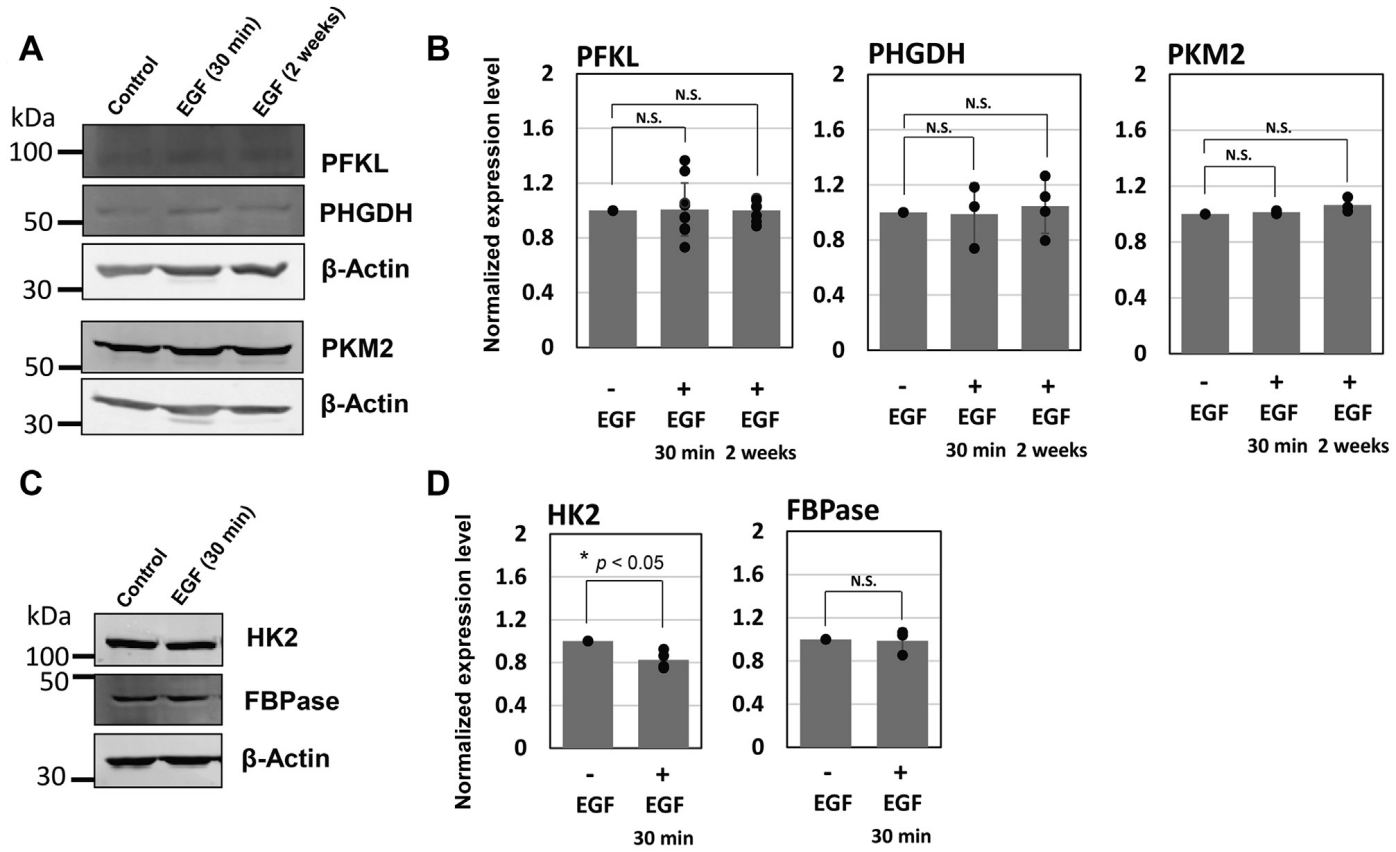
**Enzyme expression in EGF-treated Hs578T cells.** Hs578T cells with and without EGF treatment were subjected to Western blot analyses (*A* and *C*) against enzymes in glucose metabolism, including phosphofructokinase liver-type (PFKL), hexokinase II (HK2), fructose 1,6-bisphosphatase live-type (FBPase), and pyruvate kinase muscle-type 2 (PKM2), as well as phosphoglycerate dehydrogenase (PHGDH) in serine biosynthesis. Note that one of the Western blots of a load control, β-actin, in (*A*) is the same blot as shown in Figure 1*B* because PFKL and PHGDH in (*A*) and the proteins in Figure 1*B* were blotted from the same gel. *B* and *D*, expression levels of PFKL, PHGDH, PKM2, HK2, and FBPase were normalized based on load controls (β-actin), respectively. The error bars represent the SDs of at least three independent experiments. Statistical analyses were performed using two-sample two-tailed *t* test. ∗*p* < 0.05. N.S.; not significant. EGF, epidermal growth factor.

We further performed Western blot analyses to gain an insight of whether the spatial transition between large- and medium-sized glucosomes by EGF–ERK1/2 signaling pathways was because of the potential changes of expression levels of relevant enzymes. In this analysis, we measured the expression levels of enzymes in glucose metabolism, including PFKL, hexokinase II, fructose 1,6-bisphosphatase, and pyruvate kinase muscle-type 2, as well as an enzyme in serine biosynthesis, PHGDH. Regardless of the duration of EGF treatment (*i.e.*, 30 min or 2 weeks), their expression levels remained unchanged (Fig. 5), except for hexokinase II being slightly reduced (Fig. 5, *C* and *D*). This result provides another layer of our understanding that real-time association and dissociation of the enzymes into/from glucosomes lead spatial transition between large and medium sizes at subcellular levels, rather than the changes of their expression levels.

## Discussion

We have previously revealed that noncancerous human breast Hs578Bst cells do not exhibit large-sized glucosomes (1), suggesting that large-sized glucosomes we observed in Hs578T and other cancer cells may be cancer-relevant and thus differentially regulated in cancer cells. At the same time, we have found that EGF, which appear to promote glycolysis-derived serine biosynthesis (7, 16), increases the population of Hs578T cells showing large-sized glucosomes (1). After screening 20 protein kinases in this work that may be associated with EGF-mediated metabolic regulation in cancer cells, we identify that only ERK1/2 is activated by the treatment of EGF (Fig. 1). This data indicates that ERK1/2 mediate a cellular communication between extracellular EGF and the upregulation of large-sized glucosomes in Hs578T cells. In addition, our pharmacological inhibition of ERK1/2 by SCH772984 and genetic knockdown of ERK1/2 using shRNAs corroborate each other to strengthen our finding of the regulatory association between the EGF–ERK1/2 signaling pathways and glucosome assemblies in cancer cells. However, it is not clear yet what kind of effectors ERK1/2 may be associated with next in its downstream to promote and maintain large-sized glucosomes at a certain level in Hs578T cells. Nevertheless, our study here strongly supports that EGF–ERK1/2 signaling pathways are responsible for regulating large-sized glucosomes to promote glycolysis-driven serine biosynthesis in human cancer cells.

In addition, our work here affirms that glucosome assemblies are indeed phase-separated biomolecular condensates in human cells. First, we have previously demonstrated that glucosome assemblies are spatially formed in the cytoplasm of human cells by dynamic partitioning of metabolic enzymes (1), indicating no membrane surrounding the glucosome assemblies. Second, due to the dynamic nature of glucosome assemblies in live cells, their fusion and fission processes are indeed constantly occurring and robustly observed although their preferential sizes are closely regulated by unidentified factors yet. In this work, we disclose a mechanism of EGF action in the regulation of large-sized glucosomes in cancer cells. Third, our Western blot analyses have now revealed that the treatment of EGF in Hs578T cells does not influence on the expression levels of enzymes involved in glucosome assemblies (Fig. 5) although it promotes the formation of large-sized glucosomes. It means that the spatial transition between large- and medium-sized glucosomes we have observed in live cells (Figs. 2 and 3) is independent of the expression levels of glucosome components rather due to the dynamic nature of phase-separated condensates. Collectively, this work reveals not only that EGF–ERK1/2 signaling pathways are closely associated with the spatial dynamics of large-sized glucosome particularly in cancer cells but also that dynamic association and dissociation of glucosome assemblies are a concentration-independent process in living human cells.

Furthermore, our work here provides a regulatory mechanism to strengthen our hypothesis that large-sized glucosome assemblies are functionally responsible for coordinating glycolysis-derived serine flux at subcellular levels. Previously, breast cancer cells have shown to promote serine biosynthetic flux though the activation of PHGDH that catalyzes the first step of serine biosynthesis (16, 25). However, a regulatory mechanism that activates PHGDH in breast cancer cells has not been identified yet. Meanwhile, the effect of EGF is recently linked to the activation of ERK1/2 to promote serine biosynthetic flux from glycolysis but only in colorectal cancer (7). Therefore, our study here discloses that the EGF–ERK1/2 signaling pathway is a regulatory mechanism that governs glycolysis-derived serine flux in breast cancer cells through the spatial regulation of large-sized glucosomes.

Collectively, we provide compelling evidence that cancer-relevant large-sized glucosomes are spatially and functionally associated with EGF–ERK1/2 signaling pathways in human breast cancer Hs578T cells. ERK1/2 activation is generally critical to the development, proliferation, differentiation, and migration of cancer cells (3, 4). Despite the successful development of potent ERK1/2 inhibitors (21), emerging drug resistance and dose-limiting side effects bring questions for their efficacies (26). Therefore, we envision that our study may provide another layer of a therapeutic insight for human breast cancer treatment.

## Experimental procedures

### Materials

Construction of mEGFP-tagged PFKL (PFKL-mEGFP) was previously described (1). Epidermal growth factor was purchased from Sigma (Cat# E4127) and dissolved in 1× PBS (Corning, Cat# 21-040-CV, 0.1 mg/ml). Then, 30 ng/ml EGF was supplemented to the Roswell Park Memorial Institute 1640 medium (RPMI 1640, Mediatech, Cat# 10-040-CV) that contains 10% dFBS. Note that FBS (Sigma, Cat# F2442) was dialyzed with Spectra Por 12 to 14,000 MWCO as previously described (27, 28). ERK1/2 inhibitor (SCH772984, Selleckchem, Cat# S7101) and Akt inhibitor X (Sigma, Cat# 124020) were dissolved in DMSO (Sigma, Cat# 276855).

### Cell culture and transfection

Human triple-negative breast carcinoma Hs578T (HTB-126) cell line was obtained from the American Type Culture Collection (ATCC). The cells were maintained in the RPMI 1640 medium supplemented with 10% dFBS and 50 μg/ml gentamycin sulfate (Corning, Cat# 61-098-RF) in a HeraCell CO_2_ incubator (37 °C, 5% CO_2_, and 95% humidity) (27). The cells were also verified to be free of *mycoplasma* contamination by employing the Universal *Mycoplasma* Detection Kit (ATCC, Cat# 30-1012K) and authenticated by ATCC’s short tandem repeat profiling service. When the cells reached about 80 to 90% confluence, they were gently removed from a culture flask by replacing their growth medium with the trypsin-EDTA solution (Corning, Cat # 25-053-Cl). For live-cell imaging, the cells were plated in an antibiotic-free growth medium (*i.e.*, RPMI 1640 with 10% dFBS) on glass-bottomed 35 mm Petri dishes (MatTek). When the confluency became ∼70 to 90% the next day, transfection was carried out using Lipofectamine 2000 (Invitrogen). After 5 h of posttransfection in the Opti-MEM-I reduced serum medium (Opti-MEM-I; Gibco, Cat# 11058), the transfection medium was exchanged with the fresh antibiotic-free growth medium that does not contain phenol red (Gibco, Cat# 11835-030) and incubated in a HeraCell CO_2_ incubator (37 °C, 5% CO_2_, and 95% humidity). On the next day of imaging, the transfected cells were washed three times with an imaging solution (20 mM Hepes (pH 7.4), 135 mM NaCl, 5 mM KCl, 1 mM MgCl_2_, 1.8 mM CaCl_2_, and 5.6 mM glucose), followed by at least 1 h incubation at ambient temperature before imaging.

### Fluorescence live-cell imaging

All images were obtained using a Photometrics CoolSnap EZ monochrome CCD camera with a 60× objective lens (Nikon CFI Plan Apo TIRF, 1.45 N.A.) on a Nikon Eclipse Ti inverted C2 confocal microscope. Wide-field imaging was carried out using a set of Z488/10-HC clean-up, HC TIRF dichroic, and 525/50-HC emission filter from Chroma Technology for mEGFP detection (1, 27). Each small molecule was diluted with DMSO to achieve respective final concentrations of SCH772984 (0.5 μM) and Akt inhibitor X (13 μM). A vehicle control was also carried out using DMSO.

### Image analysis

The ImageJ processing software (National Institutes of Health) was used for glucosome size analysis as reported previously (1). Fluorescent wide-field images were processed through ImageJ using a custom script and macro that automated the counting of fluorescent particles using its built-in module, robust automatic threshold selection (RATS). In this analysis, the captured images were scaled according to the pixel size of the microscope (*i.e.*, 0.12 μm × 0.12 μm per pixel) before applying the default parameters for RATS (*i.e.*, noise threshold = 25, λ factor = 3). Once fluorescent particles were selected from an image, the particle analysis module was applied to attain both the number and the area of fluorescent particles within an image. This process was repeated for all subsequent cell images. The operator then evaluated the particle mask with the original cell images to eliminate data in which more than one particle was counted as a single particle.

### Knockdown of ERK1 and ERK2 using shRNAs

Hs578T cells were transfected with shRNAs targeting ERK1/2 (shERK1 and shERK2) and a control shRNA with a scrambled sequence (shControl_Scrambled_, CGCGAAGTCTGTACTCTTG, Addgene, Cat# 65232) (24) using Lipofectamine 2000. The plasmids expressing shERK1 and shERK2 contains the sequence of CATGAAGGCCCGAAACTAC (Addgene, Cat# 65228) and CCAGATCCTCAGAGGGTTAAA (Addgene, Cat#65229), respectively (24). After 5 h of posttransfection, puromycin (Sigma, Cat# P8833, 1 μg/ml) was added to select shRNA-transfected cells. After at least 24 h of transfection, puromycin-selected cells were washed with our imaging buffer for fluorescence live-cell imaging or harvested for Western blotting.

### Western blot analysis

Hs578T cells were cultured, harvested, and lysed in RIPA buffer (20 mM Tris–HCl (pH 7.5), 140 mM NaCl, 1% Triton X-100, 0.5% sodium deoxycholate, 0.1% SDS, and 10% glycerol) that contained protease inhibitors (Pierce, Cat# 88666) and phosphatase inhibitors (Pierce, Cat# 88667). Bicinchoninic acid assay measurement (29) was subsequently used to determine protein concentrations of cell lysates. We then prepared 10% SDS-PAGE gels and loaded 40 μg of each sample per lane. BioRad Semi Dry Transfer System was subsequently used to transfer resolved proteins to a PVDF membrane. The membrane was soaked in 5% nonfat milk that was prepared in 1× TBST (10 mM Tris (pH 7.5), 150 mM NaCl, and 0.5% Tween 20), followed by washing three times with 1× TBST. Then, a primary antibody was incubated with the membrane overnight at 4 °C. The used primary antibodies included mouse anti-phospho-ERK1/2 (1:1000, Cell Signaling Technology, Cat# 9106), rabbit anti-total-ERK1/2 (1:1000, Cell Signaling Technology, Cat# 4695), rabbit anti-PFKL (1:1000, Sigma-Aldrich, Cat# HPA018257), rabbit anti-PKM2 (1:1000, Cell Signaling, Cat# 4053(D78A4)), rabbit anti-fructose-1,6-bisphosphatase (1:1000, Sigma-Aldrich, Cat# HPA005857), rabbit anti-hexokinase II (1:1000, GeneTex, Cat# GTC111525), rabbit anti-PHGDH (1:1000, Bethyl, Cat# A304-732A), and mouse anti-β-actin antibody (1:10,000, Sigma, Cat# A5441). Subsequently, a secondary antibody that was conjugated with a near-IR dye was incubated with the membrane for 1 to 2 h at room temperature, which included donkey anti-rabbit-AlexaFluor790 (1:10,000, Jackson Immunoresearch, Cat# 711-655-152) and donkey anti-mouse-DyLight690 (1:10,000, Cell Signaling, Cat# 5470). LI-COR Odyssey Sa Near-IR imager was used to scan the membrane, and ImageStudioLite (LI-COR) was used for quantitative analysis.

### Luminex multiplexing assay

Hs578T cells were cultured in RPMI 1640 with 10% dFBS. Thirty nanograms per milliliter of EGF was supplied to the cells for 5, 10, 30, 60, and 120 min, respectively. Alternatively, Hs578T cells were maintained in the growth medium with a supplement of 30 ng/ml of EGF for at least 2 weeks. Epidermal growth factor-treated cells were then harvested and lysed at each time point with the commercially available lysate buffer (Millipore, Cat# 43-040) that contained a protease inhibitor (Thermofisher, Cat# 1861284). As a control, cells in the growth medium without EGF were harvested and lysed at each time point. In this work, several commercially available Luminex magnetic microspheres (Millipore, Cat # 46-677MAG, 46-602MAG, 48-650MAG, 48-681MAG, and 48-611MAG) were used to screen the following kinases: ERK1/2, AKT, STAT3, JNK, p70 S6K, p38, GSK3a/b, IGF1R, IR, mTOR, and SRC family kinases (Blk, Fgr, Fyn, Hck, Lck, Lyn, Src, and Yes). Protein concentrations were adjusted to 1 mg/ml using MILLIPLEX MAP Assay Buffer 2 (Millipore, Cat# 43-041). Next, 25 μl of each protein lysate was added with a mixture of 10 beads per well in 96-well plate at each trial and then shaken overnight at 4 °C at 900 rpm. Next day, the plate was placed on a magnetic plate separator (Magnetic Plate Separator, Luminex, CN-0269-01, center capture), and the supernatant was removed. The beads were then washed two times with MILLIPLEX MAP Assay Buffer 2. Subsequently, 25 μl of biotinylated antibody mixture was added to each well and incubated for 30 min at room temperature and 900 rpm in the dark. The supernatant was removed, and the beads were washed twice. Next, the beads were treated with 25 μl per well of streptavidin-phycoerythrin conjugate (Millipore Cat# 45-001H) for ∼15 min and shaken at 900 rpm at room temperature in the dark. The supernatant was removed and 25 μl of the Amplification buffer was added in each well followed by 15 min shaking at 900 rpm at room temperature in the dark. Then, the beads were washed with MILLIPLEX MAP Assay Buffer 2 twice, and the plate was immediately analyzed using Flexmap3D. For positive and negative controls, 25 μl of positive control lysates (Human embryonic kidney 293 cells, Millipore Cat# 47-233) and lysis buffer were used, respectively. Median fluorescent intensities for duplicate wells at each experiment were averaged. The data reported here is from at least three duplicated independent trials with fresh cell lysates.

### Statistical analysis

Statistical analyses of high-content imaging data were performed using GraphPad Prism 9.2. Briefly, a two-way ANOVA analysis was performed to determine statistical significance among different treatments and/or between the experimental and the control groups. Tukey’s multiple comparison tests were also carried out to determine which pairs within each size group (*i.e.*, small, medium, and large) were significantly different and where a significance takes place in the two-way ANOVA analysis. Statistical analyses of Western blot data were performed using two-sample two-tailed *t* test. Statistical significance was defined as *p* < 0.05 with a 95% confidence interval.

## Data availability

All data are presented within the article.

## Conflict of interest

The authors declare that they have no conflicts of interest with the contents of this article.
